# Safety and Clinical Efficacy of Prostatic Artery Embolization in Patients with Indwelling Urinary Catheter for Benign Hyperplasia—A Multicenter Study

**DOI:** 10.3390/diagnostics14242864

**Published:** 2024-12-19

**Authors:** Jules Pouchot, Amandine Crombé, Luc Burlet, Fadi Farah, Pierre Baseilhac, Arthur David, François Petitpierre, Rim Maaloum, Yann Le Bras, Gaele Pagnoux, Haytham Derbel, Hicham Kobeiter, Matthias Barral, Julien Frandon, Clément Marcelin, Clément Klein, Eva Jambon

**Affiliations:** 1 Service de Radiologie et Imagerie Médicale de L’adulte, Centre Hospitalier Universitaire de Bordeaux, Place Amélie Raba Léon, 33076 Bordeaux, Francef.petitpierre@lecai.fr (F.P.); rim.maaloum@chu-bordeaux.fr (R.M.); yann.lebras@chu-bordeaux.fr (Y.L.B.); clement.marcelin@chu-bordeaux.fr (C.M.); eva.jambon@chu-bordeaux.fr (E.J.); 2 SARCOTARGET Team, Bordeaux Institute of Oncology (BRIC) INSERM U1312, 33076 Bordeaux, France; 3 Department of Medical Imaging, IPI Plateform, Nîmes University Hospital, University of Montpellier, Medical Imaging Group Nîmes, IMAGINE, 30029 Nîmes, France; luc.burlet@chu-nimes.fr (L.B.); julien.frandon@chu-nimes.fr (J.F.); 4 Department of Diagnostic and Interventional Medical Imaging, Assistance Publique-Hôpitaux de Paris, Hôpitaux Universitaires Henri Mondor, Université Paris Est, 94000 Créteil, Francehaytham.derbel@aphp.fr (H.D.); hicham.kobeiter@aphp.fr (H.K.); 5 Department of Uroradiology, Hospices Civils de Lyon, Hôpital Edouard Herriot, 69003 Lyon, France; pierre.baseilhac@chu-lyon.fr (P.B.); gaele.pagnoux01@chu-lyon.fr (G.P.); 6 Department of Radiology, Nantes University Hospital, University of Medicine, 44000 Nantes, France; arthur.david@chu-nantes.fr; 7 Service de Radiologie, Assistance Publique-Hôpitaux de Paris, Hôpital Tenon, Sorbonne Université, 75970 Paris, France; matthias.barral@aphp.fr; 8 UFR Médecine, Sorbonne Université, 75006 Paris, France; 9 Service de Chirurgie Urologique, Centre Hospitalier Universitaire de Bordeaux, Place Amélie Raba Léon, 33076 Bordeaux, France; clement.klein@chu-bordeaux.fr

**Keywords:** prostate artery embolization, prostate benign hyperplasia, urinary catheter, safety, treatment outcome

## Abstract

**Background/Objectives**: This multicentric study aimed to evaluate the efficacy and safety of prostatic artery embolization (PAE) to remove indwelling urinary catheter (IUC) in patients with symptomatic benign prostatic hyperplasia (BPH). Secondary objectives were to identify features associated with post-PAE catheter-free survival (PCFS). **Methods**: All consecutive patients who underwent PAE for IUC related to BPH with a follow-up of at least 2 years (except for early death) in 6 French University Hospitals were retrospectively included. Clinical efficacy was defined as the removal of the IUC after PAE (through a trial without catheter [TWOC]) and evaluated at regular intervals. Chi-square tests, Wilcoxon tests and multivariable binary logistic regressions were utilized to investigate predictors of TWOC success. Univariable and multivariable Cox regressions were utilized to investigate predictors of PCFS in patients with TWOC success. **Results**: 140 men with IUC (median age: 82.5 years, interquartile range [IQR] = 73–88.2 years, range: 46–100) who underwent PAE between January 2017 and March 2021 were included. Initial successful catheter removal (TWOC success) following PAE occurred in 113/140 (80.7%) patients, and 3/140 (2.1%) patients encountered major complications. In patients with TWOC success, PCFS at 6 months, 1 year and 2 years were 87.5% (95%CI: 81.4–94.1), 84.4% (95%CI: 77.7–91.7) and 79% (71.3–87.4), respectively. No independent predictive factors for TWOC success and PCFS were identified. **Conclusions**: PAE should be considered as a safe option with good clinical efficacy in the short and long term for elderly and inoperable patients with IUC due to symptomatic BPH.

## 1. Introduction

Acute urinary retention (AUR) is a severe complication of benign prostatic hypertrophy (BPH), characterized by a sudden and painful inability to void voluntarily, requiring immediate bladder decompression via catheterization [1]. AUR is the most frequent cause of requiring emergency urological care and affects over 10% of men aged over 70 [2,3]. The first-line treatment usually involves a Trial Without Catheter (TWOC), where the catheter is removed after 2–3 days of daily oral alpha-blocker therapy [4]. However, the reported TWOC failure rates are as high as 49.8% [5]. Moreover, even if TWOC is initially successful, there is a recurrence rate of 50.5% within the next 5 years, necessitating additional interventions [6].

The primary remaining long-term option for patients with persistent AUR is an indwelling urinary catheter (IUC). This palliative measure significantly impacts the quality of life, restricting activities of daily living for 40% of patients [7], and affecting social activities for 44% of patients. Moreover, urinary tract infections (UTIs) are the fourth most common healthcare-associated infection in the USA, with 67% associated with IUC use, leading to approximately 35,000 catheter-associated UTIs annually [8]. These infections contribute to increased morbidity, mortality, and healthcare costs [9]. Additionally, IUCs are associated with other complications such as gross hematuria, affecting 13.5% of patients. Chronic inflammation from prolonged catheter use also increases the risk of bladder cancer [10].

Surgery remains the gold standard treatment for AUR [11]. Traditional transurethral resection of the prostate (TURP) has demonstrated effectiveness, with 82.3% of patients successfully removing their catheters [12]. More recent procedures, such as Holmium Laser Enucleation of the Prostate (HoLEP), have achieved catheter removal rates as high as 98.5% [13]. However, these surgical interventions are not without side effects. Monopolar-TURP is associated with a 29.3% complication rate, while HoLEP has an 8.8% incidence of incontinence [14,15]. Furthermore, these procedures often require general anesthesia, which poses significant risks for patients with comorbidities, leaving them in a therapeutic dilemma. As a result, alternative minimally invasive techniques, such as prostate artery embolization (PAE), have recently emerged.

The aim of PAE is to embolize the prostate arteries, inducing ischemic necrosis and shrinkage of the prostatic tissue [16]. The procedure is performed on an outpatient basis under local anesthesia using either femoral or radial access [17,18]. Numerous robust studies have already demonstrated its efficacy in patients without a catheter [19,20].

Previous studies have investigated the interest in PAE for removing IUCs in the management of BPH with a clinical efficacy ranging between 45.4% and 95.2% [21,22,23,24,25,26,27,28,29,30,31]. They showed a high level of safety, with major complications occurring in less than 5% of patients. However, many of these studies only included small subgroups of patients, with a maximum of 24 participants. Additionally, patient follow-up was inconsistent, ranging from 3 months to over 2 years. Late relapses occurring up to 43 months after PAE, with a mean follow-up of 16.2 months have been observed. Long-term clinical data on larger cohorts remain scarce, highlighting the need for multicenter studies to strengthen the scientific evidence and better inform the clinical management of this patient population.

Thus, the purpose of this study was to assess the short and long-term outcomes of PAE in patients presenting with AUR due to BPH with TWOC failure and to identify factors associated with PAE success defined as no need for re-catheterization during the post-PAE follow-up.

## 2. Materials and Methods

### 2.1. Study Design

This multicenter study was approved by the institutional review board of Bordeaux University Hospital, France (CER-BDX 2024-85). The need for written informed consent was waived due to its retrospective nature.

We included all consecutive patients from six French university hospitals (Pellegrin [Bordeaux], Nîmes, Nantes, Edouard Herriot [Lyon], Tenon [Paris] and Henri Mondor [Paris-Créteil]) between January 2017 and March 2021.

All presented with IUC and TWOC failure due to complicated BPH and were referred by their urologists to the interventional radiology departments for PAE because of failed catheter removal and unsuitable surgical options. Reasons for surgery not being pursued included high morbidity risks due to poor general health or patient refusal. It must be noted that for patients capable of making their own decisions, we provided detailed oral and written information on the benefits and risks of both embolization and surgery. In cases where patients were unable to decide, we consulted their families and the primary physician (geriatrician, general practitioner, or urologist) to facilitate a multidisciplinary decision regarding the proposed treatment.

Patients were excluded if urinary obstruction resulted from causes other than BPH, specifically proven prostate carcinoma or urethral stricture, or if urinary retention was related to neurological conditions. Of note, all patients included in the study were assessed by an urologist, and in cases where urethral stricture was suspected, a fibroscopy was systematically performed. Additionally, difficulty in catheterization (including catheter size) was considered a factor suggestive of a potential stricture. Patient follow-up was also conducted by an urologist who, in cases of clinical suspicion of urethral stricture, could perform a fibroscopy to rule out this diagnosis.

### 2.2. Definition of Patient Outcomes

The primary objective was to evaluate the safety and early and long-term clinical efficacy of PAE in this population. Secondary objectives were to identify features associated with post-PAE TWOC and catheter-free survival (PCFS).

Early clinical efficacy was defined as the successful removal of the bladder catheter within the first two months after PAE. Typically, the urinary catheter was removed in a day hospital setting, most often within the urology department. Following removal, patients were monitored until they successfully voided. A bladder scan was then performed to confirm the absence of significant post-void residual volume, ensuring safe catheter removal. Long-term clinical efficacy was defined as the sustained removal of the bladder catheter without relapse or the need for prostate surgery in patients who remained alive at least two years after PAE. Technical efficacy was defined as achieving bilateral embolization.

Complications were documented, and adverse events were assessed using the Clavien-Dindo classification. The variability in the literature suggests that minor complications are often underestimated in retrospective data collection, leading to a lack of representativeness. Consequently, only major complications were recorded due to the retrospective design.

For patients demonstrating early clinical efficacy, PCFS was defined as the duration (in months) between the initial catheter removal following PAE and any subsequent re-catheterization. Patients who died before re-catheterization were censored from this analysis.

### 2.3. PAE Procedure

#### 2.3.1. Common Steps

PAE were all performed by senior interventional radiologists according to the latest guidelines and under local anesthesia [32]. The key steps of the procedure included: (1) insertion of a 5Fr vascular sheath into either the right common femoral artery or the left radial artery; (2) cannulation of the contralateral internal iliac artery; (3) 3D rotational cone-beam CT angiography; and (4) supra-selective catheterization of the prostatic arteries. Following arteriography, embolization was performed using microspheres (300–500 μm in diameter), N-butyl cyanoacrylate glue, or a combination of both. In cases where there was a risk of non-target embolization due to anastomotic vessels, a protective coil embolization was performed. The choice of embolization agents was determined based on the experience of each center.

#### 2.3.2. End of the Procedure

The injection was halted upon observing significant reflux beyond the first 1–2 mm at the tip of the microcatheter. Vascular access was secured using occlusion devices appropriate for either radial or femoral access. After embolization, patients were monitored in a dedicated care unit and immediate complications were documented. Patients were discharged from the hospital if no complications arose. However, some patients may have been hospitalized overnight as a precaution due to poor general health or if they lacked support at home.

### 2.4. Data Collection

Data were collected through a review of medical records and imaging reports by senior radiologists from each center. All patients underwent imaging and clinical follow-up after PAE. A radiological or urological consultation was systematically planned within the first two months after PAE with an initial TWOC.

Baseline assessment included age (considered continuously and categorized as <75 years, 75–84 years, and ≥85 years), duration of IUC before PAE (considered continuously and categorized as <3 months, 3–6 months, 6–12 months, and >12 months), number of cardiovascular risk factors, contraindication for general anesthesia, the number of drug oral therapeutics before introduction of IUC, previous surgery for BPH, and treatment with antiplatelet or anticoagulants agents.

Pre-PAE prostate volume was measured with MRI or US imaging using the ellipsoid formula and considered continuously and categorized as <50 cm^3^, 50–99 cm^3^, 100–149 cm^3^, and ≥150 cm^3^.

Technical data were recorded from angiography images and radiological reports and comprised: the total duration of the 1st procedure (categorized as <1 h, 1–2 h, and >2 h), the type of embolization agent used (categorized as microparticle, glue, both, and other), the irradiation dose received, the number of PAE procedures attempted and whether bilateral PAE was obtained. In the event of PAE failure, the type of surgery performed and its details were recorded. Centers were categorized as those with fewer than 20 patients and those with ≥20 patients.

### 2.5. Statistical Analysis

Statistical analyses were performed using R (version 4.1.0, Vienna, Austria). All tests were two-tailed. A *p*-value < 0.05 was deemed significant.

Continuous variables were reported as a median interquartile range (IQR) and minimum–maximum range, or mean ± standard deviation [SD], and minimum–maximum range depending on the Shapiro–Wilk normality test, whereas categorical variables were reported as numbers and percentages.

Predictive factors for early clinical success (i.e., immediate TWOC success after PAE) were investigated using univariable chi-square tests for categorical variables and unpaired *t*-test or Mann–Whitney test for numeric variables, which was complemented with multivariable binary logistic regression. Odds ratio (ORs) with 95% confidence intervals (95%CI) were estimated.

Predictive factors for PCFS were identified using univariable and multivariable Cox proportional hazard ratio regressions. Hazard ratios (HR) with 95%CI were estimated. Characteristics with a univariable *p*-value less than 0.100 were entered in the multivariable analyses. The Kaplan–Meier method was utilized to draw the PCFS curve.

## 3. Results

### 3.1. Study Population

**Figure 1:**
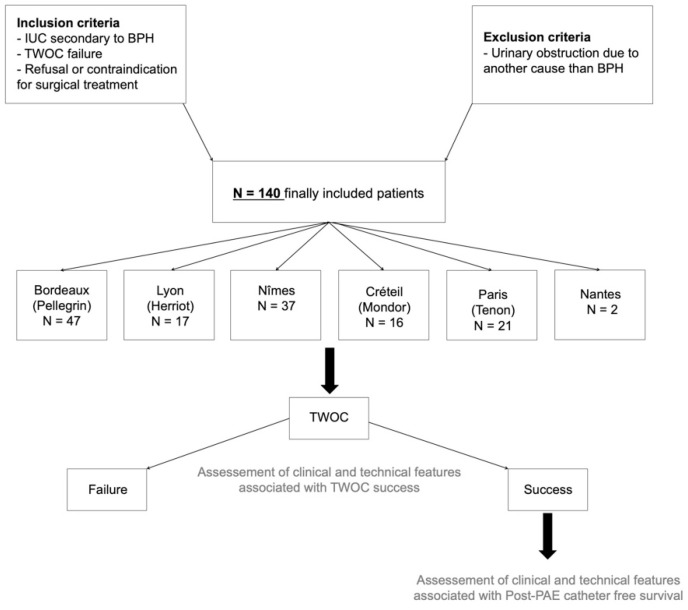
Study workflow. Abbreviations: IUC: indwelling urinary catheter; BPH: benign prostatic hyperplasia; TWOC: trial without catheter.

From January 2017 to December 2021, 140 consecutive men (median age: 82.5 years, IQR: 73–88.2 years; range: 46–100 years) with UIC secondary to BPH treated by PAE were included in six French university hospitals (Figure 1).

Four patients underwent PAE despite a confounding neurological bladder disease, or to treat an AUR caused by active prostatic carcinoma and were previously excluded.

The baseline characteristics of the patients are summarized in Table 1. The median prostate volume was 90 cm^3^ (IQR: 68–120, range: 28–350 cm^3^). The intermediate duration of IUC before PAE was 4 months (IQR: 2–6, range: 1–36 months). Forty-four patients (31.4%) were deemed unsuitable for general anesthesia.

### 3.2. Characteristics of the PAE Procedure (Table 2)

A total of 127/140 (90.7%) patients were treated with only one PAE procedure and 13/140 (9.3%) required at least two interventions.

Technical success (i.e., bilateral embolization) occurred in 102/140 (72.9%) patients after one procedure and in one additional patient after two procedures.

The median irradiation dose was 12.823 µGym^2^ (IQR: 7.439–21.503, range: 1.045–86.356 µGym^2^).

Regarding embolization agents, 100/140 (71.4%) procedures were performed with microspheres, 29/140 (20.8%) with glue (N-butyl cyanoacrylate), 9/140 (6.4%) with both, and 2/140 (1.4%) with other agents.

### 3.3. Clinical Outcomes (Table 3)

Forty (28.6%) patients died during the study follow-up.

**Table 3:** General outcome data following prostate artery embolization.

Characteristics	Patients
Length of hospital stay (days)	1 [1–1] (1–7)
TWOC success	113/140 (80.7)
Time from PAE to TWOC (days)	28 [21–36.8] (7–120)
Total no. of relapse post-TWOC success ^1^	27/113 (23.9)
No. of relapse in patients included in PCFS analysis ^2^	23/109 (21.1)
Time from TWOC to relapse (days) in relapsing patients	180 [90–420] (30–930)
Death during study follow-up	40/140 (28.6)
Probability of post-PAE catheter-free survival (PCFS)	
At 3 months post-TWOC success	92.4% (95%CI: 87.4–97.6)
At 6 months post-TWOC success	87.5% (95%CI: 81.4–94.1)
At 1 year post-TWOC success	84.4% (95%CI: 77.7–91.7)
At 2 years post-TWOC success	79% (95%CI: 71.3–87.4)

NOTE—Abbreviations: 95%CI: 95% confidence interval, PAE: prostatic artery embolization, PCFS: post-PAE catheter-free survival, TWOC: trial without catheter. Quantitative variables are expressed as median with interquartile range in brackets and range in parentheses number. Qualitative data are expressed as raw numbers and percentages in parentheses. Survival probabilities are expressed as percentages with 95%CI. ^1^: In all patients with a TWOC success. ^2^: In patients with TWOC success and available relapse status and date.

A TWOC success following PAE was reported in 113/140 (80.7%) patients. The median TWOC delay was 28 days (IQR: 21–36.8, range: 7–120 days). None of the patients were lost to follow-up but the date of re-catheterization following TWOC success was missing in four patients resulting in 109/113 (96.4%) patients being included in the PCFS sub-analysis.

Regarding the PCFS analysis, 23/109 (21.1%) patients required re-catheterization despite an initial TWOC success. Of those, 10/23 (43.5%) patients were treated with surgery (three with TURP and seven with HoLEP).

The median follow-up in patients who did not relapse was 777.5 days after the TWOC success (IQR: 692–991.5, range: 2–2642 days).

In patients who relapsed after an initial TWOC success, the median delay before a new IUC was 180 days (IQR: 90–420, range: 30–930 days).

**Figure 2:**
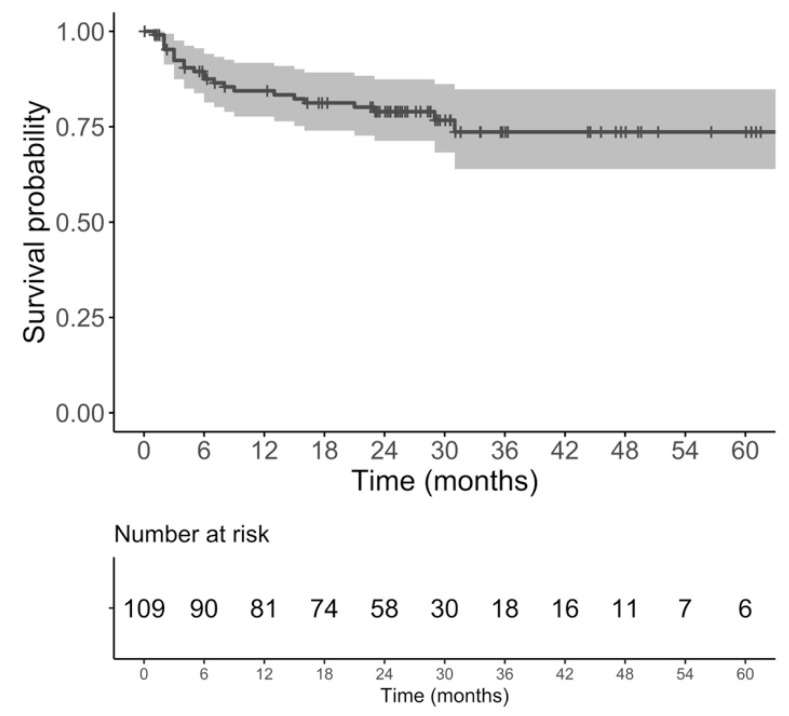
Kaplan–Meier curve of catheter-free survival following prostate artery embolization (PCFS) in patients with a successful trial without catheter (TWOC). The gray area corresponds to the 95% confidence interval.

Using Kaplan Meier analysis, the PCFS probabilities were 92.4% (95%CI: 87.4–97.6) at 3 months, 87.5% (95%CI: 81.4–94.1) at 6 months, 84.4% (95%CI: 77.7–91.7) at 1 year, and 79% (95%CI: 71.3–87.4) at 2 years (Figure 2).

Overall, 3/140 (2.1%) patients encountered major complications (grade > II according to the Clavien-Dindo classification), which consisted of temporary penile ulcerations in one patient treated by topical treatment and two hematomas of the femoral approach which required an endovascular treatment. No severe infectious complications, such as sepsis or prostatic abscess, were observed.

### 3.4. Assessment of Features Associated with TWOC and PCFS

Table 4 shows the univariable analysis of the features associated with TWOC. In the univariable analysis, no significant association was found although a trend toward lower rates of TWOC success was observed in patients with longer duration of IUC before PAE (Chi-square test *p*-value = 0.0944—OR = 1.94, 95%CI: 0.65–5.81 [*p* = 0.2346] for a duration of 6–12 months; and OR= 3.64, 95%CI: 0.66–20.01 [*p* = 0.1370] for a duration longer than one year, using the 0–3 months group as reference).

Table 5 shows the univariable analysis of the features associated with PCFS. None of the initial clinical and technical features were associated with PCFS.

The center and volume of patients per center were not associated with TWOC success and PCFS.

## 4. Discussion

This observational study presents the largest multicenter cohort to date evaluating the short- and long-term outcomes of patients with IUC due to BPH treated with PAE. The results aligned with previous studies, demonstrating the efficacy and safety of PAE for this specific population. However, none of the pre-PAE clinical features or pre-PAE technical aspects were associated with TWOC success or PCFS.

Concerning the efficacy of PAE in this population, the technical success rate was 72.9%, slightly below the usual rates seen in the general population. Typically, the rate of unilateral PAE is below 10%, with a maximum reported rate of 18.6% [27]. This disparity may stem from the increased procedural complexity in patients with indwelling urinary catheters (IUCs), primarily due to the prevalence of atherosclerosis, which leads to tortuosity of the iliac vessels.

Despite the technical challenges posed by atherosclerosis in this population, embolization procedures did not appear to be unduly prolonged. Over half of the PAE interventions (57.1%) lasted ≤2 h, compared to a mean procedure time of 130 min reported by Frandon et al. in a study of 383 patients, including 99 with IUC [21]. Additionally, this multicenter study confirms that PAE can be safely performed without discontinuation of antiplatelet or anticoagulant therapy, supporting existing knowledge [19,33,34]. The length of hospital stay was also recorded, underscoring that PAE is an outpatient procedure even for these comorbid patients. The median hospital stay in our study was 1 day.

The initial TWOC success rate was 80.7%, aligning with a recent analysis of 88 patients that reported an 82% success rate [23]. In the same study, 16% of patients experienced a relapse requiring re-catheterization within an average period of 16.2 months. In our study, we observed a recurrence rate of about 20% at a median follow-up of 6 months after PAE.

The incidence of major complications in our study was 2.1%, underscoring the safety of PAE, particularly in a population with a higher proportion of fragile patients compared to the general population. This statistic is similar to the tolerability profile observed in a large multi-institutional cohort of 383 patients included in the study by Frandon et al., which reported 1.2% severe adverse effects [21]. Notably, no urinary infections were observed in our study, in contrast to previous publications [28,29].

Our secondary objectives were to identify clinical or technical factors contributing to TWOC failure and poor PCFS after initial TWOC success. No center effect on short- and long-term efficacy was observed. This could be attributed to the fact that the selected treatment centers were institutions with significant expertise in prostate embolization, likely having already surpassed the initial learning curve.

One hypothesis suggested that prostatic revascularization might occur, potentially resulting from the formation of collateral arterial pathways [25,35]. These additional vascular afferents may also have been pre-existing, particularly in patients with atherosclerosis. In this context, cardiovascular comorbidity was the single independent variable associated with PAE failure in two previous studies, but this association was not confirmed in the study by Marchi et al., which corroborates our findings [20,21]. This discrepancy might be attributed to the characteristics of our predominantly atherosclerotic patient population, as only 19.9% of our patients were unaffected, potentially leading to limited statistical power.

No association was identified between technical success and clinical success. Although bilateral PAE has been found superior to unilateral PAE in terms of clinical outcomes in some previous articles [36], our results are consistent with more recent observations [21,23].

Other variables have been suggested as predictive factors of PAE success in earlier studies with large cohorts, such as an age of under 65 years. However, age did not influence long-term clinical success in our work, consistent with other past studies [21,23].

Additionally, prostatic volume did not influence long-term clinical success. The median prostatic volume in this study was 90 cm^3^. These results strengthen the findings of several previous studies that demonstrated the benefits of PAE for large prostatic glands [37,38]. Nevertheless, the efficiency of PAE on smaller prostates remains unclear.

We also investigated the duration of IUC before PAE and its relation to catheter-free survival. It could be hypothesized that an indwelling catheter might cause detrusor contraction dishabituation, resulting in impaired bladder function. Although Yuan et al. suggested a relationship between IUC duration before PAE and the ability for catheter removal, no statistical correlation was observed in our study regarding catheter-free survival [38].

Moreover, no additional predictive factors for long-term PAE success were identified in our study, aligning with the existing literature [21,23].

The observed recurrence rate was about 20% at two years, and half of the relapsing patients were treated with surgery, illustrating that undergoing PAE does not preclude subsequent surgical intervention.

This study has limitations. As a retrospective analysis, it lacked potentially predictive features such as body mass index, performance status, International Prostate Symptom Score, quality of life questionnaires, and changes in prostate volume following PAE. Hence, it was not possible to confirm a significant reduction in prostate volume. However, according to the literature, long-term studies reported prostate volume reduction ranging from −6.8 to −39 mL after PAE in the general population [20,39,40,41,42]. Additionally, 22.1% of patients were censored due to early deaths, and the timing for performing TWOC varied widely, ranging from 1 to 8 weeks. While urinary retention is a known transient complication of PAE, occurring in approximately 5% of patients as reported in a recent meta-analysis, this is often due to initial edema that may exacerbate urethral compression [43]. However, there is no evidence to suggest that the timing of TWOC affects the success of catheter removal.

## 5. Conclusions

To conclude, this study suggests that PAE is a safe and efficient therapeutic alternative in patients with IUC due to BPH, especially in case of contraindication or refusal of surgical procedures. Future improvements in PAE outcomes and the assessment of its safety and efficacy will require better-defined selection criteria, standardization of practices, and regular evaluations in the setting of prospective clinical trials.

## Figures and Tables

**Table 1:** Clinical characteristics of the study population.

Characteristics		Patients
**Age (years)**		82.5 [73–88.2] (46–100)
**Prostate volume (cm^3^)**		90 [67.8–120] (28–350)
**Imaging modality to assess prostatic volume**		
	Ultrasonography	75 (53.6)
	MRI	65 (46.4)
**Previous oral treatments for BPH**		
	None	25 (17.9)
	Monotherapy	53 (37.9)
	Bi- or tritherapy	62 (44.3)
**Previous surgical treatment for BPH**		
	TURP	4 (2.8)
	Open prostatectomy	1 (0.6)
	HoLEP	1 (0.6)
**Duration of IUC before PAE (months)**		4 [2–6] (1–36)
No. of cardiovascular risk factors *		
	0	17 (12.1)
	1	26 (18.6)
	2	50 (35.7)
	3	33 (23.6)
	4	14 (10)
**Anti-thrombotic treatments**		
	None	43 (30.7)
	Antiplatelet therapy	38 (27.1)
	Anticoagulant therapy	41 (29.3)
	Anticoagulation + Antiplatelet therapy	18 (12.9)
**Contraindication for general anesthesia**		44 (31.4)

NOTE—Abbreviations: BPH: benign prostate hypertrophy; HoLEP: Holmium laser enucleation of the prostate; IUC: indwelling urinary catheter; No.: number; PAE: prostate artery embolization; TURP: Transurethral resection of the prostate. Quantitative variables are expressed as median with interquartile range in bracket and range in parentheses number. Qualitative data are expressed as raw numbers and percentages in parentheses. * Cardiovascular risk factors are high blood pressure, diabetes, hypercholesterolemia, and personal history of cardiovascular disease.

**Table 2:** Per-procedure technical data.

Characteristics		Patients
**Number of procedures**		
	1	127 (90.7)
	≥2	13 (9.3)
**Duration of the procedure §**		
	<1 h	31 (22.1)
	1–2 h	49 (35)
	>2 h	53 (37.8)
**Irradiation dose §**		12,823 [7439–21,503] (1045–296,356)
**Embolization Agent**		
	Microspheres	100 (71.4)
	Glue	29 (20.8)
	Microsphere + Glue	9 (6.4)
	Other	2 (1.4)
**Technical success (bilateral embolization)**		
	No	102 (72.9)
	Yes, after 1 procedure	37 (26.4)
	Yes, after ≥2 procedures	1 (0.7)

NOTE—Quantitative variables are expressed as median with interquartile range in brackets and range in parentheses number. Qualitative data are expressed as raw numbers and percentages in parentheses. §: Irradiation dose and duration of the procedure correspond to the first prostate artery embolization.

**Table 4:** Assessment of associations between clinical and technical characteristics and the success of a trial without catheter (TWOC i.e., early clinical efficacy) following prostate artery embolization (PAE).

Characteristics		TWOC Success	TWOC Failure	*p*-Value
**Center**				0.7513
	Bordeaux (Pellegrin)	38/113 (33.6)	9/27 (33.3)	
	Lyon (Edouard Herriot)	15/113 (13.3)	2/27 (7.4)	
	Nîmes	31/113 (27.4)	6/27 (22.2)	
	Créteil (Henri Mondor)	12/113 (10.6)	4/27 (14.8)	
	Paris (Tenon)	16/113 (14.2)	5/27 (18.5)	
	Nantes	1/113 (0.9)	1/27 (3.7)	
**No. of patients included in center**				1.000
	<20 included patients	28/113 (24.8)	7/27 (25.9)	
	≥20 patients	85/113 (75.2)	20/27 (74.1)	
**Age (continuous, years) §**		82 [73–87] (46–100)	85 [76.5–90] (63–95)	0.1589
**Age groups**				0.1935
	<75 years	35/113 (31)	7/27 (25.9)	
	75–84 years	40/113 (35.4)	6/27 (22.2)	
	≥85 years	38/113 (33.6)	14/27 (51.9)	
**No. of cardiovascular risk factors**				0.8537
	0	15/113 (13.3)	2/27 (7.4)	
	1	20/113 (17.7)	6/27 (22.2)	
	2	39/113 (34.5)	11/27 (40.7)	
	3	27/113 (23.9)	6/27 (22.2)	
	4	12/113 (10.6)	2/27 (7.4)	
**Antithrombotic treatment**				0.6590
	None	32/113 (28.3)	11/27 (40.7)	
	Antiplatelet	32/113 (28.3)	6/27 (22.2)	
	Anticoagulant + antiplatelet	15/113 (13.3)	3/27 (11.1)	
	Anticoagulant	34/113 (30.1)	7/27 (25.9)	
**Contraindication for general anesthesia (yes)**		34/111 (30.6)	10/27 (37)	0.6815
**Prostate volume (continuous, cm^3^) §**		98 [72–120] (28–350)	90 [60–125] (40–250)	0.4905
**Prostate volume (groups)**				0.3833
	<50 cm^3^	9/113 (8)	1/27 (3.7)	
	100–150 cm^3^	40/113 (35.4)	6/27 (22.2)	
	50–100 cm^3^	48/113 (42.5)	16/27 (59.3)	
	>150 cm^3^	16/113 (14.2)	4/27 (14.8)	
**Embolization agent**				0.6349
	Microparticles	80/113 (70.8)	20/27 (74.1)	
	Glue	24/113 (21.2)	5/27 (18.5)	
	Microparticles + Glue	8/113 (7.1)	1/27 (3.7)	
	Other	1/113 (0.9)	1/27 (3.7)	
**No. of PAE procedures**				0.4573
	1	101/113 (89.4)	26/27 (96.3)	
	≥2	12/113 (10.6)	1/27 (3.7)	
**Time from PAE to TWOC (continuous, days) §**		4 [2–6] (1–36)	5 [2–7] (1–36)	0.1685
**Time from PAE to TWOC (groups)**				0.0952
	< 3 months	34/113 (30.1)	7/27 (25.9)	
	3–6 months	50/113 (44.2)	7/27 (25.9)	
	6–12 months	25/113 (22.1)	10/27 (37)	
	>12 months	4/113 (3.5)	3/27 (11.1)	
**Technical success**		83/113 (73.5)	19/27 (70.4)	0.9342

NOTE. Tests are the Chi-square test, except for §, which corresponds to unpaired Wilcoxon tests. Quantitative variables are expressed as median with interquartile range in brackets and range in parentheses number. Qualitative data are expressed as raw numbers and percentages in parentheses.

**Table 5:** Assessment of associations between clinical and technical characteristics and post-PAE catheter-free survival (PCFS).

Characteristics		No. at Risk	No. of Events	PCFS Probability at 6 Months	PCFS Probability at 2 Years	Log Rank *p*-Value	HR (95%CI)	Cox *p*-Value
**Center**								
	Bordeaux (Pellegrin)	37	10	80.13 (67.98–94.46)	71.23 (57.64–88.03)	0.6514	reference	reference
	Lyon (Edouard Herriot)	14	3	100 (100–100)	85.71 (69.21–100)		0.64 (0.17–2.32)	0.4925
	Nîmes	30	4	96.67 (90.45–100)	89.23 (78.41–100)		0.42 (0.13–1.34)	0.1439
	Créteil (Henri Mondor)	11	2	80.81 (60.04–100)	80.81 (60.04–100)		0.75 (0.16–3.4)	0.7041
	Paris (Tenon)	16	4	78.57 (59.77–100)	69.84 (48.82–99.91)		1.19 (0.37–3.81)	0.7751
	Nantes	1	0	100 (100–100)	100 (100–100)		0 (0–Inf)	0.998
**No. of patients included in center**								
	<20 included patients	26	5	92.15 (82.27–100)	83.56 (70.01–99.73)	0.7061	reference	reference
	≥20 patients	83	18	85.99 (78.63–94.03)	77.55 (68.66–87.6)		1.21 (0.45–3.27)	0.7039
**Age (continuous, years) §**		-	-	-	-	-	1.01 (0.96–1.05)	0.7973
**Age groups**								
	<75 years	34	6	90.91 (81.61–100)	81.48 (69.12–96.05)	0.6593	reference	reference
	75–84 years	37	7	84.9 (73.53–98.03)	84.9 (73.53–98.03)		1.24 (0.42–3.69)	0.7002
	≥85 years	38	10	86.6 (76.32–98.26)	71.52 (57.88–88.37)		1.58 (0.57–4.35)	0.3779
**No. of cardiovascular risk factors**								
	0	12	1	100 (100–100)	91.67 (77.29–100)	0.4797	reference	reference
	1	20	4	90 (77.77–100)	84.71 (70.17–100)		2.65 (0.3–23.75)	0.383
	2	39	10	83.46 (72.19–96.48)	74.16 (60.87–90.34)		3.94 (0.5–30.78)	0.1913
	3	27	7	84.44 (71.52–99.7)	71.95 (56.26–92.02)		4.1 (0.5–33.38)	0.187
	4	11	1	90 (73.2–100)	90 (73.2–100)		1.29 (0.08–20.7)	0.8572
**Antithrombotic treatment**								
	None	30	5	96.55 (90.13–100)	85.54 (73.37–99.73)	0.2413	reference	reference
	Antiplatelet	32	5	86.54 (75.09–99.73)	82.78 (70.08–97.77)		1.05 (0.3–3.62)	0.9419
	Anticoagulant + antiplatelet	14	2	91.67 (77.29–100)	83.33 (64.7–100)		1.04 (0.2–5.37)	0.9615
	Anticoagulant	33	11	78.2 (65.14–93.88)	67.91 (53.25–86.61)		2.37 (0.82–6.82)	0.1102
**Contraindication for general anesthesia**								
	No	74	15	87.22 (79.75–95.39)	79.68 (70.69–89.81)	0.5789	reference	reference
	Yes	33	8	87.27 (76.35–99.75)	74.93 (60.2–93.26)		1.27 (0.54–3.01)	0.5827
**Prostate volume (continuous, cm^3^) §**		-	-	-	-	-	0.99 (0.99–1)	0.2703
**Prostate volume (groups)**								
	<50 cm^3^	9	2	88.89 (70.56–100)	76.19 (52.08–100)	0.4696	reference	reference
	100–150 cm^3^	38	9	88.72 (78.89–99.77)	76.8 (63.86–92.35)		1.11 (0.24–5.12)	0.8968
	50–100 cm^3^	47	11	81.98 (71.4–94.12)	74.36 (62.31–88.75)		1.16 (0.26–5.26)	0.8435
	>150 cm^3^	15	1	100 (100–100)	100 (100–100)		0.26 (0.02–2.84)	0.268
**Embolization agent**								
	Microparticles	77	15	90.55 (84.13–97.47)	81.6 (73.01–91.2)	0.4477	reference	reference
	Glue	24	5	78.26 (63.1–97.07)	78.26 (63.1–97.07)		1.16 (0.42–3.19)	0.7762
	Microparticles + Glue	7	3	83.33 (58.27–100)	50 (22.46–100)		2.61 (0.75–9.07)	0.1298
	Other	1	0	100 (100–100)	100 (100–100)		0 (0–Inf)	0.9976
**No. of PAE procedures**								
	1	97	21	87.24 (80.75–94.26)	78.97 (70.96–87.9)	0.9294	reference	reference
	≥2	12	2	90 (73.2–100)	78.75 (56.41–100)		0.94 (0.22–4.01)	0.9322
**Time from PAE to TWOC (continuous, days) §**		-	-	-	-	-	0.98 (0.87–1.1)	0.6922
**Time from PAE to TWOC (groups)**								
	< 3 months	33	6	89.6 (79.1–100)	77.54 (63.11–95.27)	0.7558	reference	reference
	3–6 months	47	11	87.09 (77.95–97.29)	80.38 (69.65–92.76)		1.09 (0.4–2.96)	0.8595
	6–12 months	25	6	83.33 (69.68–99.66)	73.46 (57.17–94.39)		1.29 (0.42–4.02)	0.6547
	>12 months	4	0	100 (100–100)	100 (100–100)		0 (0–Inf)	0.9974
**Technical success**								
	Yes	80	17	84.61 (76.97–93.01)	79.1 (70.47–88.79)	0.9931	reference	reference
	No	29	6	95.83 (88.16–100)	77.31 (61.61–97.02)		0.99 (0.39–2.52)	0.9842

NOTE—Abbreviations: 95%CI: 95% confidence interval, HR: hazard ratio, no.: number, PAE: prostate artery embolization, TWOC: trial without catheter. §: for continuous variables, only univariable Cox regressions could be performed.

## Data Availability

The raw data and R code used to produce the statistical results and figures are not publicly available but can be provided by the corresponding author upon reasonable request.
